# Femtosecond tunable solitons up to 4.8  µm using soliton self-frequency shift in an InF_3_ fiber

**DOI:** 10.1038/s41598-022-19658-8

**Published:** 2022-09-23

**Authors:** Jean-Christophe Gauthier, Michel Olivier, Pascal Paradis, Marie-Frédérique Dumas, Martin Bernier, Réal Vallée

**Affiliations:** 1 Center for Optics, Photonics and Lasers (COPL), Université Laval, Québec, G1V 0A6 Canada; 2 Département de Physique, Cégep Garneau, Québec, G1S 4S3 Canada

**Keywords:** Applied optics, Lasers, LEDs and light sources

## Abstract

A tunable ultrashort soliton pulse source reaching up to 4.8 µm is demonstrated based on a 2.8 µm femtosecond fiber laser coupled to a zirconium fluoride fiber amplifier followed by a small core indium fluoride fiber. This demonstration is extending by 300 nm the long wavelength limit previously reported with soliton self-frequency shift (SSFS) sources based on fluoride fibers. Our experimental and numerical investigation highlighted the spectral dynamics associated with the generation of highly redshifted pulses in the mid-infrared using SSFS enhanced by soliton fission. This study is intended at providing a better understanding of the potential and limitations of SSFS based tunable femtosecond fiber sources in the 3–5  µm spectral range.

## Introduction

Since the first modelocking experiments in the 60s^1^ and the advent of the Ti:Sapphire laser^2,3^, ultrafast laser pulses have found a significant number of applications in a wide variety of areas. In parallel to this, one of the new hotbed of laser-based applications is the mid-infrared (MIR) spectral range, which is conveniently defined in the context of optical fiber as spanning between 2.5–20  µm^4^. Including most fundamental molecular oscillations and offering an atmospheric transparency window between 3–5  µm, the MIR has been subject to growing scientific efforts over the last 20 years aimed at developing new laser systems and techniques to harness its strong application potential^5–8^. Accordingly, free-space broadband parametric sources were developed but such sources usually require complex alignment and are often considered as bulky and costly systems. To circumvent this, great effort has been dedicated to the development of ultrafast lasers emitting directly in the MIR region, such as fiber lasers around 2.7–3.1 µm^9–15^ and around 3.55 µm^16,17^, as well as Fe:ZnSe lasers centered around 4.4  µm^18–20^. While Fe:ZnSe lasers can produce mJ pulses with duration as short as 150 fs^20^, they still require multiple free-space components and complex alignment. Moreover, the Fe:ZnSe crystal needs cryogenic cooling to operate around 4.4  µm which further complicates its widespread utilization^21^. On the other hand, ultrafast MIR fiber lasers have the advantage of a relatively simple and robust architecture with superior beam quality ($\hbox {M}^2$ $\sim$ 1.1), and good performance in terms of pulse duration and peak power^15,22,23^. Nevertheless, up to now, their limited wavelength coverage is leaving most of the 3–5  µm spectral region untouched.

An efficient and convenient alternative to produce ultrashort pulses at tunable wavelengths is via the soliton self-frequency shift (SSFS) process, which is driven by the nonlinear Raman effect^24,25^. Simply put, ultrashort pulses with a sufficiently broad spectrum and enough peak power to trigger stimulated Raman scattering will undergo a progressive redshift as they propagate in an optical fiber. This technique was notably used to produce tunable soliton pulses between 2.8 and 3.6  µm with 160 fs pulse duration and 200 kW of peak power using a fiber amplifier to redshift seed pulses from a mode-locked 2.8  µm fiber laser^26^. More recently, a similar approach using a Cr:ZnS oscillator coupled to a 2.5 cm long passive ZBLAN fiber also allowed to produce sub-100 fs pulses tunable from 2.3 to 3.85  µm with $\sim$ 35 kW peak power in the main soliton^19^. Tunable solitons from 3 to 3.8 µm were also obtained in cascaded $\hbox {Er}^{3+}$-doped and $\hbox {Dy}^{3+}$-doped fluoride fiber amplifiers^27^. Until 2022, the longest wavelength achieved with this technique was 4.3  µm as demonstrated by Tang et al., producing 100 fs pulses with peak powers ranging between 20 and 75 kW^28^. It used 550 fs (1.3  µJ) pulses from an erbium-doped fiber chirped pulse amplification (CPA) system at 1.55  µm shifted successively through SSFS in a polarization-maintaining rod-type photonic crystal fiber (PCF) and in an indium fluoride fiber using free-space coupling. This result was then improved in 2022 by Tiliouine et al. using a similar approach. Pulses from a fiber CPA laser (1  µJ, 1970 nm, 765 fs pulses at 1 MHz) were launched in a cascade of silica Large Mode Area (LMA) fiber, ZBLAN LMA fiber, another ZBLAN fiber and an $\hbox {InF}_3$ fiber, resulting in a shifted soliton centered at 4.5 µm^29^.

Although this demonstration convincingly outlined the potential of MIR SSFS sources, it also showed the limitations of this approach to further extend the spectral coverage without either relying on even more energetic ultrashort seed pulses or adding more fibers to an already complex multi-stage assembly. Other approaches were proposed to achieve similar or higher wavelength shift, but only numerical results were reported to support them^30,31^.

In the present study, we experimentally demonstrate SSFS tuning of femtosecond pulses up to 4.8  µm using a mode-locked 2.8  µm fiber laser coupled to a zirconium fluoride fiber amplifier fusion-spliced to a smaller core size indium fluoride fiber, effectively extending the previously reported long wavelength edge by 300 nm. Numerical simulations outline the significant impact of the soliton fission occurring at the beginning of the small-core indium fluoride fiber to enhance the spectral shift. By giving access to femtosecond pulses covering most of the 3–5  µm region from a simpler fiberized system, we expect to enable new opportunities for applications such as molecular spectroscopy, material processing and pump-probe experiments.

## Experimental results

**Figure 1:**
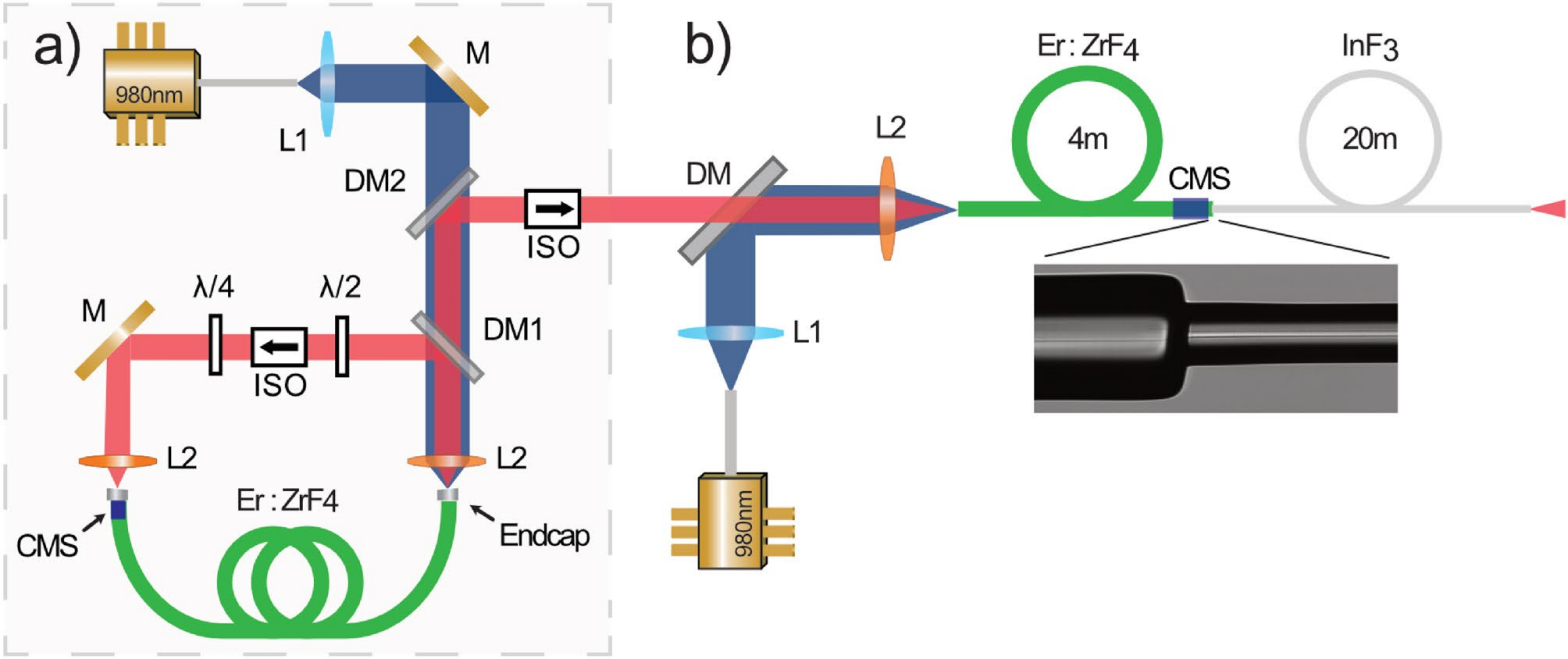
Schematic of the experimental setup. The spectral tuning is selected via the 980 nm pump power which controls the amplification process, and thus the achievable redshift. (**a**) 2.8  µm mode-locked fiber laser (**b**) SSFS module. Abbrev. *M*: Gold Mirror; *L1*: Silica; *L2*: AR-coated ZnSe; *CMS*: Cladding Mode Stripper; *DM*: Dichroic Mirror; *ISO*: Isolator.

Our experimental setup first relies on a ring cavity fiber laser emitting 440 fs pulses at 2.8  µm and 4 nJ pulse energy (@ 57.9 MHz, 230 mW) based on the nonlinear polarization evolution mode-locking technique. More details about this mode-locked oscillator can be found elsewhere et al.^22,26^. This seed pulse train is subsequently directed toward two optical fiber stages where SSFS is taking place, hereafter labeled the *in-amplifier SSFS* and the *soliton fission SSFS* stages. These stages are associated with the propagation in an active erbium-doped fluorozirconate (Er:$\hbox {ZrF}_4$) fiber and a passive indium fluoride ($\hbox {InF}_3$) fiber respectively, as shown in Fig. 1.

### In-amplifier SSFS

**Figure 2:**
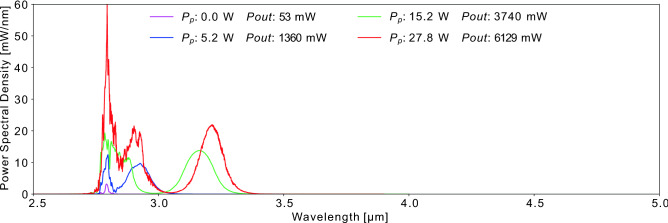
Power spectra at the output of the 4m active fiber for various values of Pp, the pump power. Pout is the output power. Seed pulse corresponds to the pink curve.

In the first stage, the seed pulses are launched in a 15/240-260  µm double-clad erbium-doped (7 mol%) fluoride fiber amplifier pumped in the first cladding by a 980 nm pump diode. Due to different coupling losses, only 53 mW of the original 230 mW of power is actually launched into the first SSFS stage. In this segment, the pulses benefit from the erbium gain at 2.8  µm in fluoride fibers^32^ and start to experience SSFS as the pump power is increased. The output spectrum after the 4 m active fiber is presented in Fig. 2.

Typical of in-amplifier SSFS, a redshifted soliton emerges as the pump power increases, later followed by secondary solitons formed by the amplification of the pulse remnants at 2.8  µm. Eventually, the spectral shift begins to saturate around 3.2  µm mostly due to the selected fiber length. While a longer amplifier could slightly boost the spectral shift, this would come at the expense of more secondary solitons^26^. Hence, an undoped fiber is preferred to enhance the spectral shift in the second stage of the setup.

### Soliton fission SSFS

**Figure 3:**
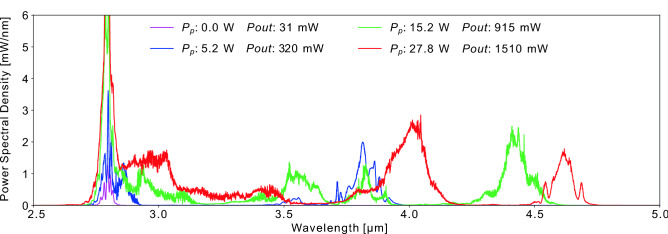
Evolution of the power spectra at the output of the passive 20 m long $\hbox {InF}_3$ fiber for increasing values of Pp.

In order to push the soliton toward longer wavelengths, the active fiber is fusion spliced to a 20 m segment of 7.5/125  µm $\hbox {InF}_3$ fiber provided by *Le Verre Fluoré*. By reducing the fiber core diameter by a factor of two, the nonlinearity sharply increases which causes the creation of a higher order soliton (N $\approx$ 2.7)^33^. Soliton fission thus ensues resulting in the creation of several fundamental solitons that each experience SSFS individually throughout their propagation. The measured spectra at the output of the $\hbox {InF}_3$ fiber for pump powers matching those of Fig. 2 are shown in Fig. 3.

**Figure 4:**
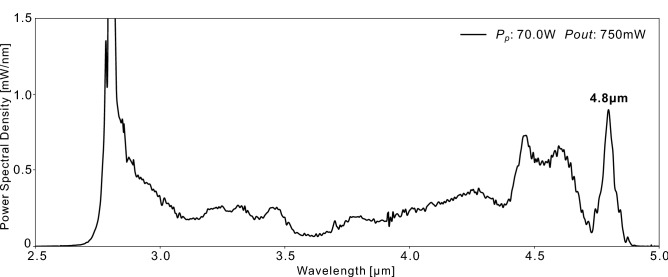
A redshidfted soliton at 4.8  µm is achieved when pumping at 70 W with a 25% duty cycle.

Thanks to the soliton fission and the SSFS process in the $\hbox {InF}_3$ fiber, a soliton at 4.62  µm is achieved at the maximum available pump power of 27.8 W. To reach the maximum redshift achievable with this setup configuration, we then switched the 30 W pump diode with a 70 W pump diode at the same wavelength. However, we had noticed in previous experiments that the polymer coating of the $\hbox {InF}_3$ fiber tended to burn several centimeters after the splice due to the fiber core mismatch and strong absorption of these polymers between 3 and 4  µm. To protect the fiber from thermal failure near the splice at high power, we operated the 70 W pump diode in quasi-continuous regime (QCW), with 25 ms square pulses at a 10 Hz repetition rate, for a duty-cycle of 25%. The pump pulse duration was chosen long enough to mimic CW operation for the seed pulses. The achieved spectra can be seen in Fig. 4.

At the maximum pump power of 70 W, solitons get shifted up to 4.8  µm, containing 7.5% of the total output power (56.25 mW, based on the area under the curve). This would correspond to an average power of 225 mW in CW operation if we take into account the duty-cycle of 25%. Unfortunately, we could not directly measure the pulse duration or temporal profile of the 4.8  µm spectral peak because no autocorrelator was available at this wavelength. The minimal pulse duration can nonetheless be estimated assuming a transform limited $\hbox {sech}^2$ spectral shape (time-bandwidth product = 0.315) for a single soliton. From the 50 nm full-width at half-maximum (FWHM), a duration of 485 fs is inferred. Given the repetition rate of 57.9 MHz and the 25% duty-cycle, the pulse energy would be of $\sim$ 4 nJ.

### Influence of $\hbox {InF}_3$ fiber geometry

**Figure 5:**
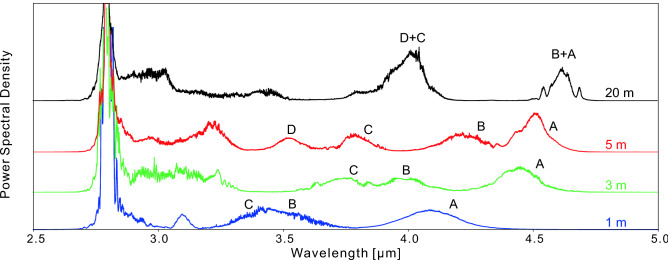
Power spectra in obtained after four different lengths of the $\hbox {InF}_3$ fiber and for Pp = 27.8 W. The curves are offset for a better visualization.

To better understand the spectral evolution in the $\hbox {InF}_3$ fiber, we progressively reduced the fiber length from 20 m to 5 m, 3 m, and 1 m. These experiments were made with the 30 W pump diode to facilitate the comparison. The resulting spectra are shown in Fig. 5. As expected, a longer fiber led to a larger redshift. Still, a soliton peaking near 4.1  µm was achieved with only 1 m of $\hbox {InF}_3$ fiber. As the fiber length was increased, different spectral peaks (labelled as *A*, *B*, etc.) associated with different solitons underwent gradual spectral shifts toward longer wavelengths. Then, the shifting of the most shifted soliton is slowed down beyond 4.5  µm due to the increase of both the fiber dispersion and mode area.

**Figure 6:**
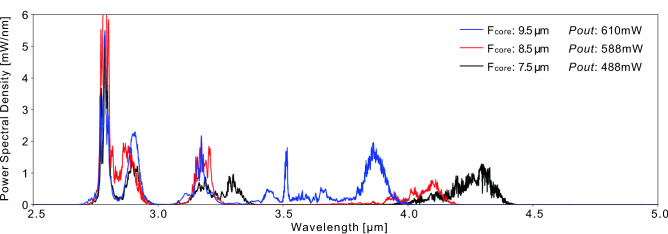
Power spectral density for three different fiber core diameters. All curves were taken at 15.2 W of incident pump power. The different output powers result from the increasing splice losses caused by the fiber dimension mismatch as the core size is reduced.

The core diameter of the $\hbox {InF}_3$ fiber was also varied to assess its effect on the spectrum. The 7.5  µm core fiber was replaced with 8.5 and 9.5  µm core diameter fibers. Since we did not want to unnecessarily cut the 8.5 and 9.5  µm fibers, we decided to use their full length of 30 and 32 m respectively. Because the difference in spectral shift between 20 and 30 m is expected to be negligible, we compare the spectra obtained with each fiber in Fig. 6 for a same pump power of 15.2 W. Such relatively low pump power was preferred in order to ensure no damage would occur at the fusion splice. Also, the amplifier length was of 3 m instead of 4 m as in the previous experiment. Thanks to the higher nonlinearity granted by the smaller fiber core, a larger spectral shift is obtained with the 7.5  µm core $\hbox {InF}_3$ fiber despite its length was 10 m less shorter.

## Simulations

### The simulator

A numerical simulator was developed in order to better understand the soliton dynamics behind our experimental results. This simulator is described in more detail in the “Methods” section. It considers the single-pass propagation of the signal within the Er:$\hbox {ZrF}_4$ fiber followed by the $\hbox {InF}_3$ fiber, taking into account the spectral dependence of the dispersive and nonlinear fiber parameters. The input signal is representative of the experimental input described above, i.e. a train of hyperbolic secant pulses with a peak power of 1830 W and duration of 440 fs, at 57.9 MHz. To get better a agreement with experimental results, white noise with a bandwidth of 0.4  µm centered on 2.8  µm and with an average power of 30 mW was added to the input. This noise could arise from different contributions: amplified spontaneous emission from the laser source or from the amplifier itself, as well as possible feedback coming from different parasitic reflections in the system and amplified by the gain fiber.

### General behavior of the system

**Figure 7:**
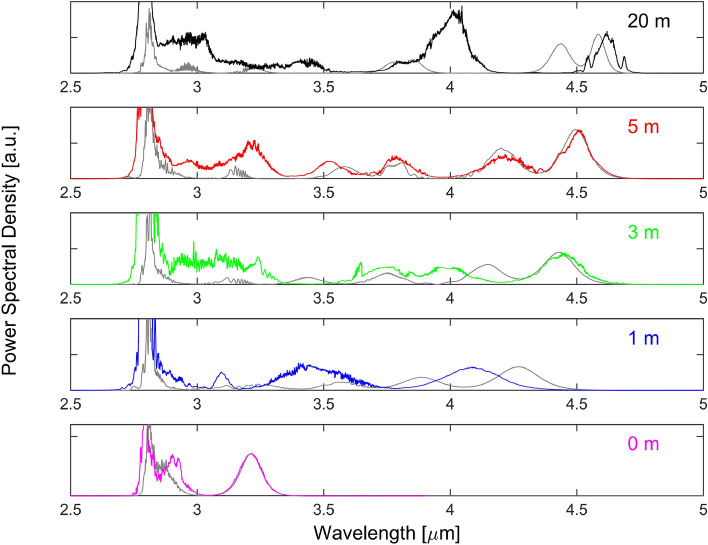
Simulated (gray) and experimental (colors) spectra for different propagation distances in the $\hbox {InF}_3$ fiber. The case labeled 0 m corresponds to the output of the Er:$\hbox {ZrF}_4$ fiber.

A first run of simulations was done for a pump power of 27.8 W. The spectra obtained at the input of the $\hbox {InF}_3$ fiber and at different positions along this fiber are shown in Fig. 7 in comparison with the experimental spectra reproduced from Fig. 5. We first note that the spectra at the output of the Er:$\hbox {ZrF}_4$ fiber (labelled 0 m in the figure) is in very good agreement with the result of Fig. 2. The power filling factor of the pump $\Gamma _p$ has a huge impact on the output of the gain fiber. Careful tuning of its value allows to tune the spectral shift of the first soliton to fit the experimental data, i.e. to obtain a spectral peak at 3.21  µm. As discussed below, a second soliton is formed within the amplifier. By adjusting the amount of white noise added at the input, we could control its spectral shift to a certain extent. By choosing an average power of 30 mW for this noise, the second soliton was shifted to approximately 2.88  µm, slightly below the experimental wavelength of the second soliton at 2.90  µm. The simulated spectra at different positions in the $\hbox {InF}_3$ fiber also show good concordance with the experimental results although their exact shape could not be faithfully reproduced for the whole set of data. As for the most shifted soliton, it varies from 4.3 to 4.6  µm as the fiber length is increased from 1 m to 20 m compared to 4.1 to 4.6  µm from the experiment. The spectral shift within the first meter of the $\hbox {InF}_3$ fiber is thus slightly overestimated by the simulations while in the rest of the propagation it happens at a slower rate. This result is not surprising since the nonlinear parameters $n_2$ and $f_R$ of this fiber are not precisely known and were thus adjusted (see section “Methods”) to better fit the results of the 5 m propagation.

**Figure 8:**
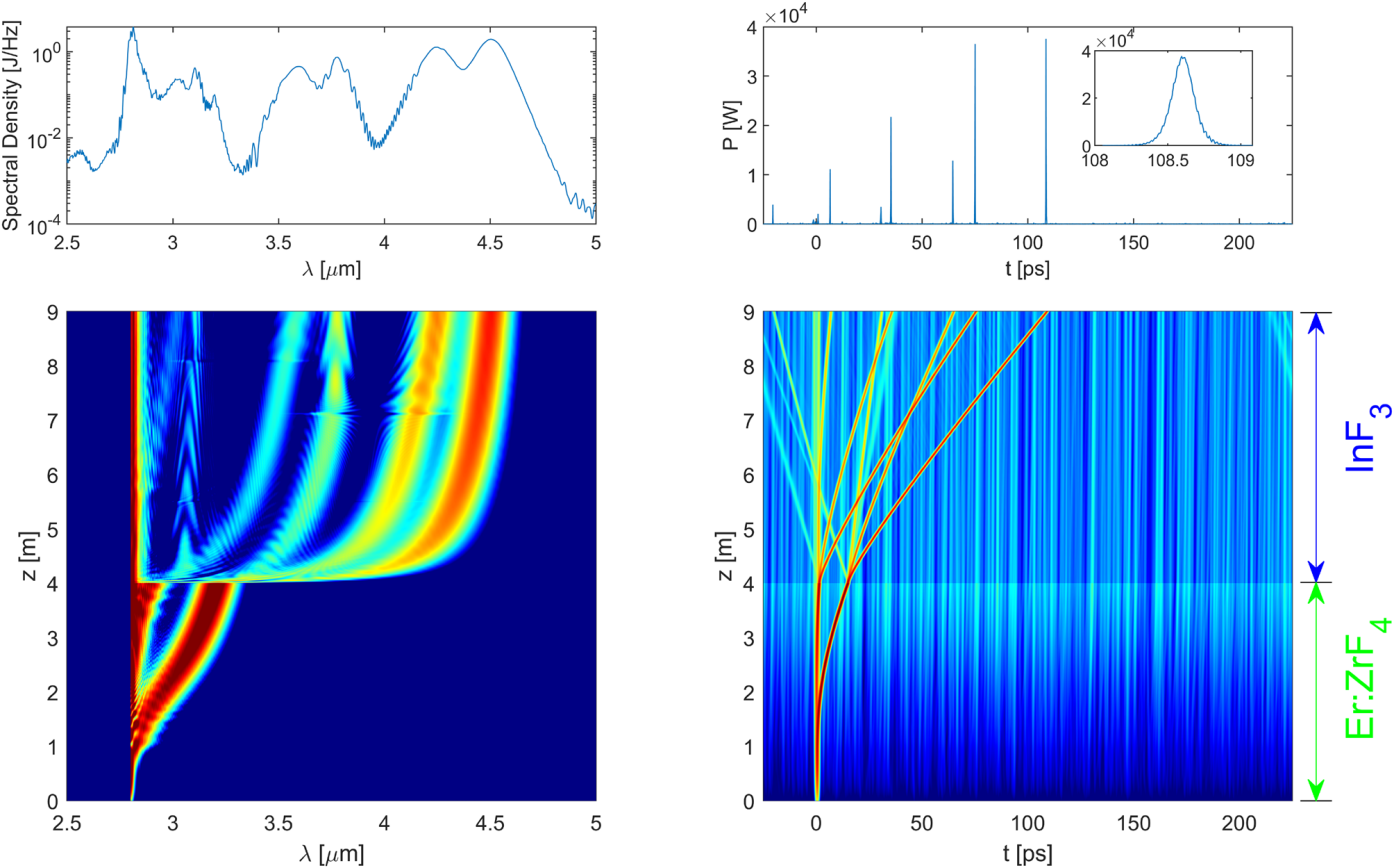
Propagation in the 4 m Er:$\hbox {ZrF}_4$ fiber and the 5 m $\hbox {InF}_3$ fiber. Top left: the spectrum at the output of the $\hbox {InF}_3$ fiber. Top right: the temporal signal at the output of the $\hbox {InF}_3$ fiber. Note that the pulses appear as vertical lines due to the size of the temporal window compared to the width of a single pulse. The inset is a zoom on the most shifted soliton to show its hyperbolic secant profile with a FWHM of 170 fs. Bottom left and right: evolution of the spectrum and the temporal signal along the fibers on a log scale. The spectrum was convoluted to be representative of the 2 nm resolution of the optical spectrum analyzer. The temporal signal was convoluted to broaden the soliton trajectories and make them appear more clearly (otherwise they would appear as thin lines barely visible on the graph).

**Figure 9:**
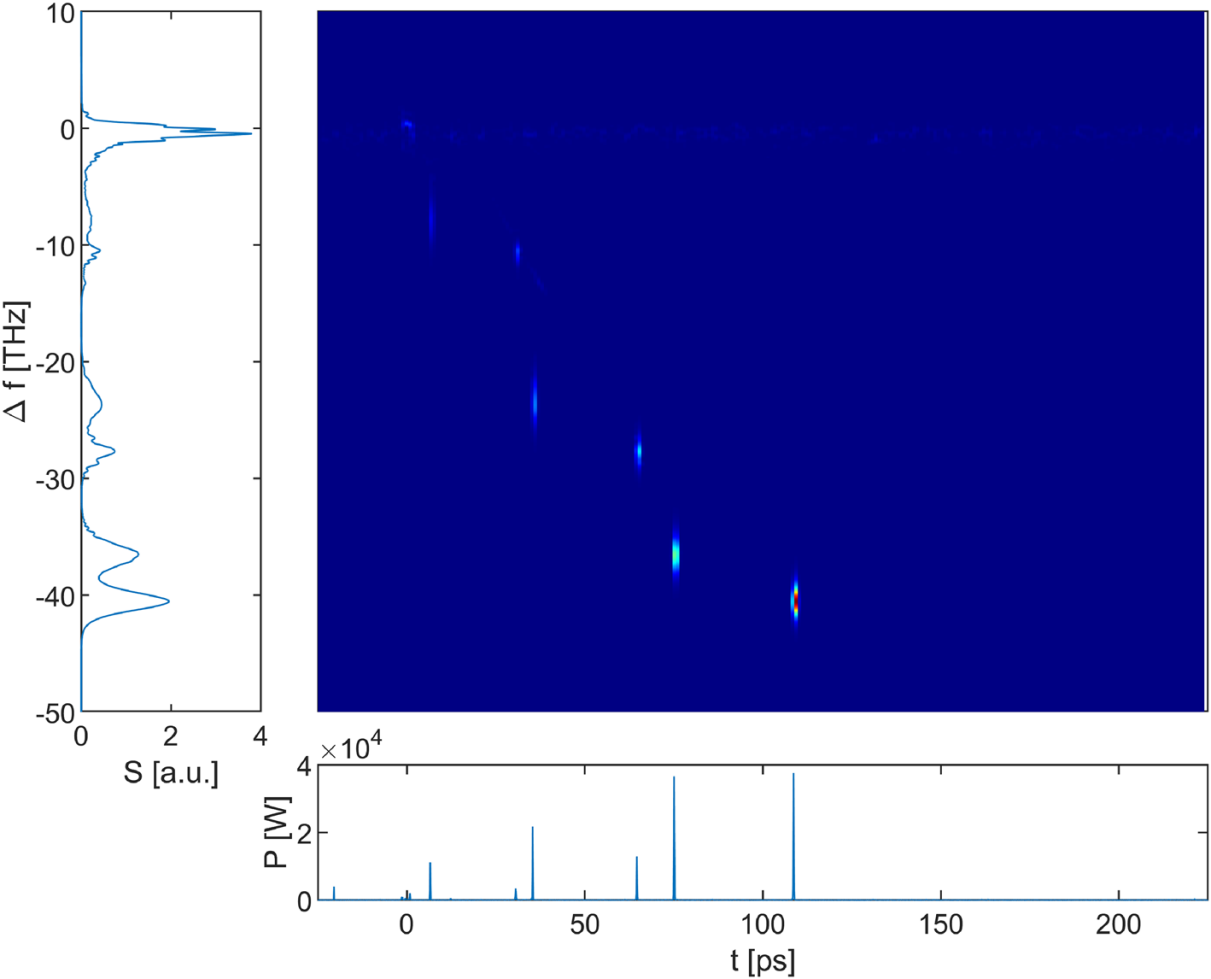
Spectrogram at the output of the system.

Figure 8 shows the parallel evolution of the spectrum and the temporal trace in the case of the 5 m propagation distance in the $\hbox {InF}_3$ fiber. Figure 9 shows the corresponding spectrogram obtained at the output of the system. The evolution of the signal within the Er:$\hbox {ZrF}_4$ fiber corresponds to a description given in a previous article^34^. As expected, the low energy input soliton is first amplified by the erbium gain. Its peak power and duration evolve adiabatically to follow the energy increase. As its peak power increases, the soliton undergoes an increasing SSFS and eventually its spectral contents leave the spectral gain bandwidth of the erbium ions. At this point, the soliton pursues its evolution in a way similar to what would occur in a passive fiber. Its wavelength continues to increase and, because of the dispersion of the fiber, the group velocity of the soliton changes as well. As it accelerates, the soliton leaves behind some dispersive waves, the so-called pulse remnants. Because those remnants are within the gain bandwidth, they are amplified and eventually transform into a second soliton that starts to undergo a SSFS^35^. Two solitons are thus present at the input of the $\hbox {InF}_3$ fiber as clearly seen on the evolution of the temporal signal at the bottom right of Fig. 8. The first one is at 3.21  µm and the second one at 2.88  µm as seen on the evolution of the spectrum at the bottom left of Fig. 8. It is important to recall that the number of solitons generated by the amplifier is governed by the available gain in the amplifier and the rate at which the SSFS occurs in the gain fiber. We also notice that the presence of white noise amplified in the Er:$\hbox {ZrF}_4$ fiber shows up as a background noise in the temporal evolution trace. In the spectrum evolution, this component manifests itself as the peak near 2.8  µm that remains throughout the propagation.

At the splice between the two fibers, the splice losses are approximately 40%. Nevertheless, both solitons entering the $\hbox {InF}_3$ fiber corresponds to a higher-order soliton ($N > 2$) due to the small core and the large nonlinearity of the $\hbox {InF}_3$ fiber. They are thus subjected to a large spectral broadening due to self-phase modulation (SPM) within the first few tens of centimeters in this fiber. Then, solitonic fission ensues due to the impact of higher-order dispersive and nonlinear effects, splitting the higher-order solitons in 2–3 fondamental solitons. In this example, the most shifted amplifier generated soliton results in the 4.5 and 3.78 µm solitons at the end of the fiber, whereas the other amplifier soliton splits in two solitons i.e. one at 4.25  µm and another one at 3.6 µm along with dispersive waves in each case. Clearly, the fiber interface and the resulting soliton fission plays a major role in the overall wavelength shift since the most shifted soliton is already shifted beyond 4.0  µm in the first 30 cm of propagation. After fission is completed, each fundamental soliton generated is subjected to SSFS until it reaches the output of the fiber. We notice that this shift is gradually slowed down along the fiber. The reason for this behavior is that as the soliton shifts to longer wavelengths, the dispersion of the fiber becomes more and more anomalous as seen in Fig. 14b and the nonlinearity of the fiber decreases due to an increasing mode-field diameter. Accordingly, due to the soliton area theorem, the soliton peak power also decreases significantly. Moreover, the losses in the $\hbox {InF}_3$ fiber increase exponentially at wavelengths above 4  µm, reducing the peak power even more. This behavior was discussed on the theoretical level recently^36^. In fact, this phenomenon is the motivation for using a cascade of several optical fibers with different properties to generate an important wavelength shift, an approach already discussed by different groups^26,28,30,35^. Looking at the spectrogram, we conclude that we get 4 main solitons at the output of the passive fiber, shifted respectively by − 40.1 THz (4.50  µm), − 36.4 THz (4.25  µm), − 27.6 THz (3.78  µm) and − 23.9 THz (3.60  µm).

### Experimental validation of the soliton fission interpretation

**Figure 10:**
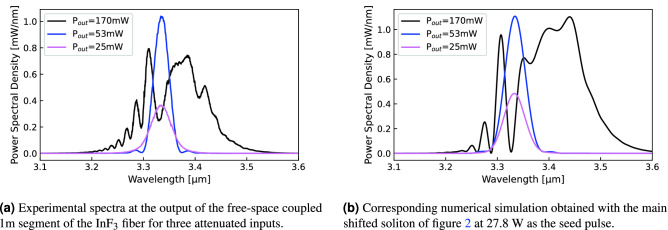
Adding neutral optical density (OD) filters before the $\hbox {InF}_3$ fiber allows to demonstrate the interference effect between the main soliton and its remnants arising as the input power is increased. $\hbox {P}_{{out}}$ corresponds to the spectrally integrated output power over 3.1–3.8 μm.

**Figure 11:**
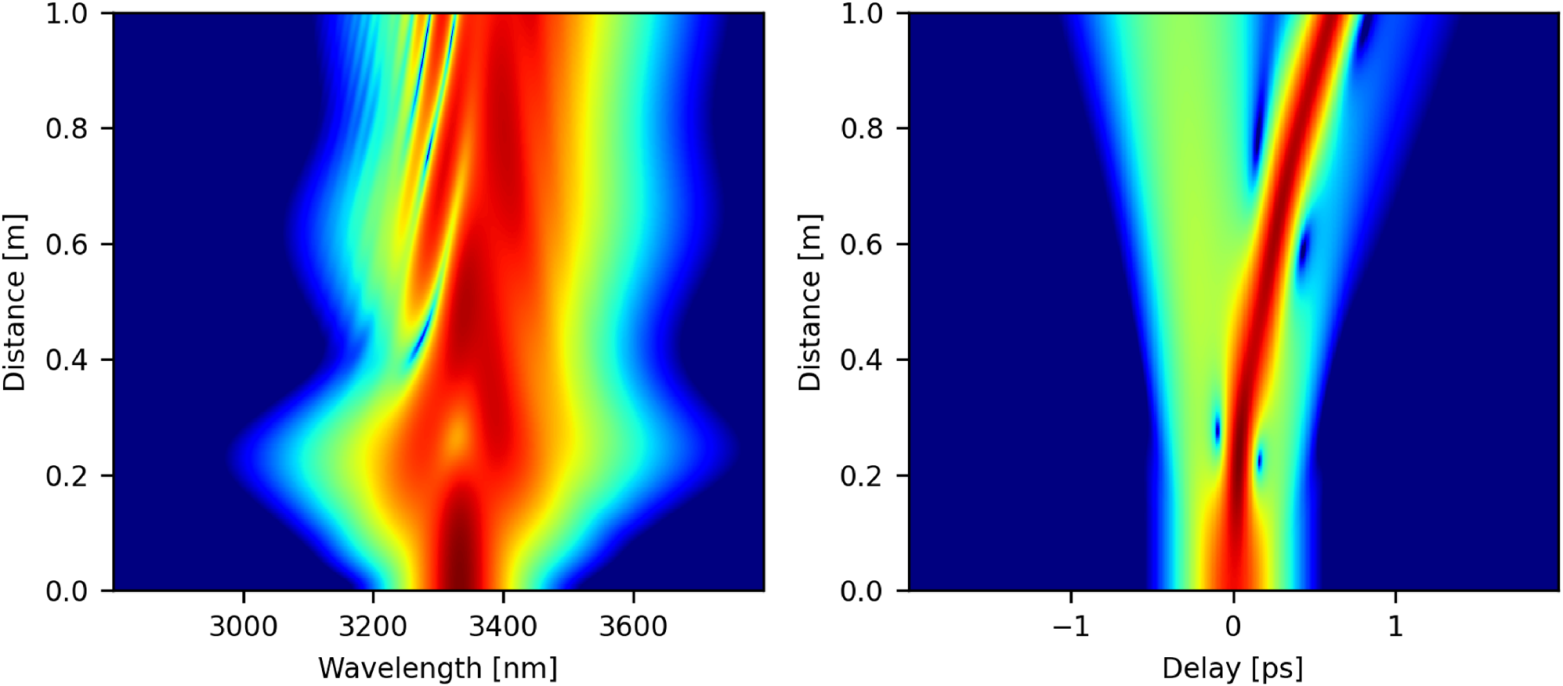
Propagation of the main soliton in the case $\hbox {P}_{{out}}$ = 170 mW (Fig.10b) within the first meter of the $\hbox {InF}_3$ fiber.

To experimentally demonstrate that soliton fission is occurring at the very beginning of the $\hbox {InF}_3$ fiber, we replaced the fusion splice between the amplifier and the $\hbox {InF}_3$ fiber by free-space coupling. We then placed neutral optical density filters between the amplifier and a 1 m segment of $\hbox {InF}_3$. A filter blocking wavelengths below 3  µm was also placed to better discriminate the spectral data. The amplifier pump power was set to 27.8 W (see Fig. 2). The measured spectra at the output of the segment of 1 m of the $\hbox {InF}_3$ fiber are shown in Fig. 10a for three different values of the output power $\hbox {P}_{{out}}$ spectrally integrated over 3.1–3.8 µm. At low injected seed power corresponding to $\hbox {P}_{{out}}$ = 25, 53 mW, the pulse has a smooth $\hbox {sech}^2$ shape typical of a fundamental soliton. However, once a certain peak power threshold is reached, the pulse starts to break apart as shown by the black curve of Fig. 10a. Numerical simulations of Fig. 10b reproduce quite accurately the fission process, using the main shifted soliton of Fig. 2 at 27.8 W as a seed pulse. In particular, the interference between the main soliton and its remnants is apparent. The pulse spectral and temporal evolution over 1 m propagation in the $\hbox {InF}_3$ fiber is also shown in Fig. 11.

### Optimization of the system

**Figure 12:**
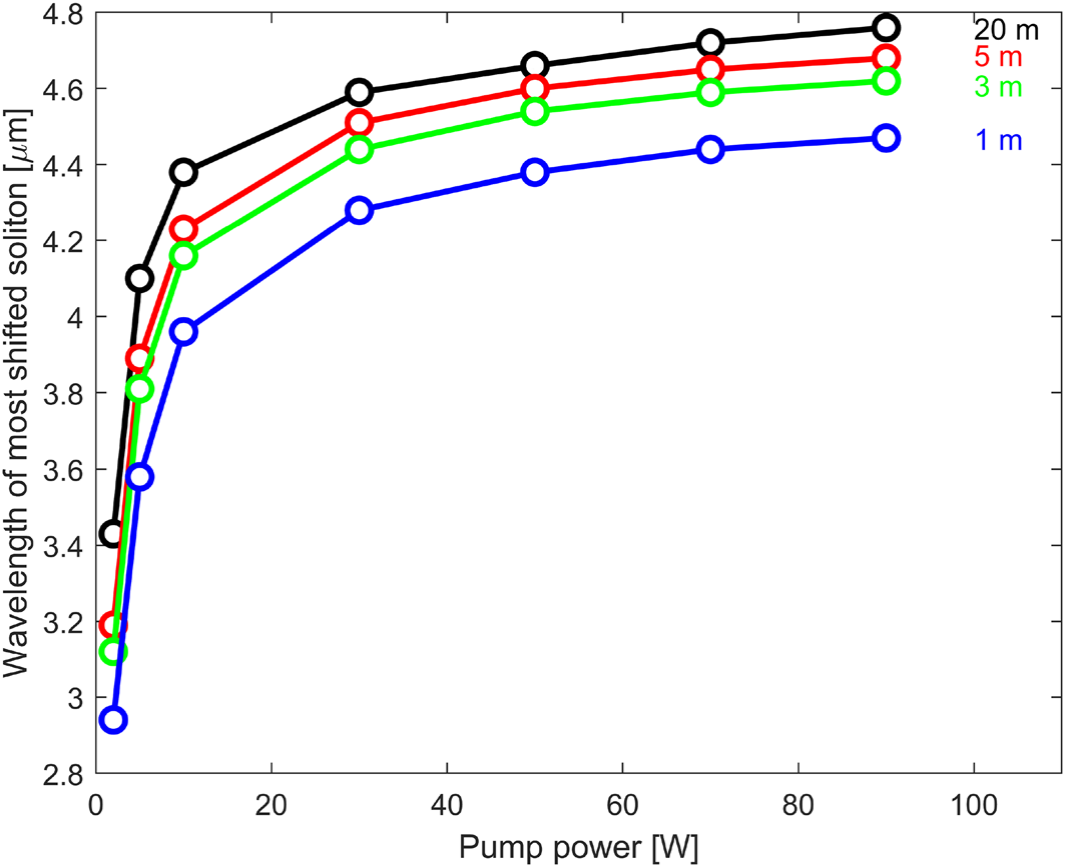
Even at high pump powers, the wavelength of the most shifted soliton does not go beyond 4.8  µm in simulation for 7.5  µm core diameter $\hbox {InF}_3$ fiber lengths of 1, 3, 5 and 20 m.

In the case of a 5 m $\hbox {InF}_3$ fiber and a pump power of 27.8 W we obtained a soliton shifted to 4.50  µm. We have seen that most of the spectral shift occurs almost immediately after the soliton fission process, while continuing at a slower rate afterwards. Simulations were performed to evaluate if this performance in terms of wavelength shift could be improved. Figure 12 shows the wavelength of the most shifted soliton as a function of pump power and $\hbox {InF}_3$ fiber length. Increasing the pump power and fiber length do result in a larger wavelength shift, but this behavior seems to saturate when the pump power reaches about 40 W. In fact, increasing the pump power will lead to the generation of more amplifier solitons, but will not affect their peak power significantly. Thus, the solitons formed after fission will not undergo a significantly larger shift. Moreover, the presence of more amplifier solitons undergoing soliton fission will crowd the spectral content at intermediate wavelengths, resulting in a supercontinuum-like spectrum. Likewise, increasing the fiber length above 5 m does not lead to a large improvement in the wavelength shift either. This is consistent with our interpretation that the soliton fission in the first meter of passive fiber is responsible for the major portion of the wavelength shift. The subsequent self-frequency shift saturates quickly for the reasons discussed previously. Nevertheless, we see that a wavelength shift up to $\sim$ 4.8  µm is approximately the best that could be done under realistic conditions, showing that our experimental setup with a 70 W pump power (Fig. 4) was close to our largest simulated spectral shift.

**Figure 13:**
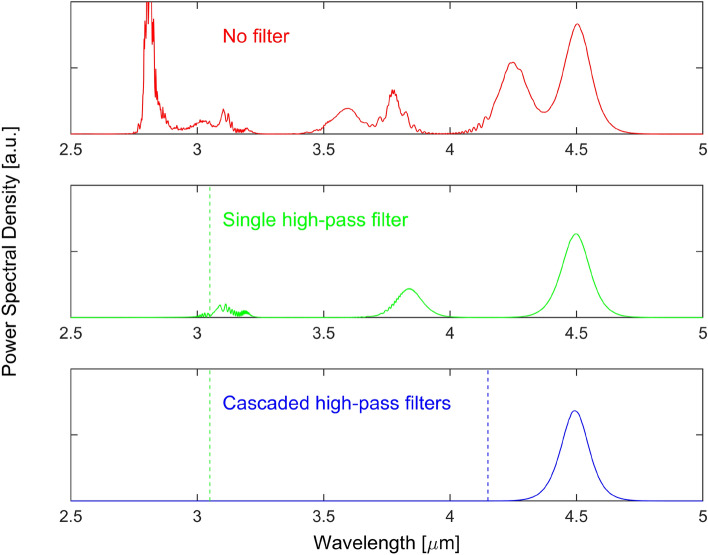
The impact of spectral filtering on the output signal: no filter (top), a single high-pass filter ($\lambda > 3.05$  µm, green dash line) between the Er:$\hbox {ZrF}_4$ and the $\hbox {InF}_3$ fibers (middle), a cascade of two filters, the first high-pass filter between the Er:$\hbox {ZrF}_4$ and the $\hbox {InF}_3$ fibers followed by a second high-pass filter ($\lambda > 4.15$  µm, blue dash line) at the output (bottom).

An aspect that could be detrimental for some applications is the presence of undesired spectral content at the fiber output. The generation of several amplifier solitons followed by the fission of each one of them in the passive fiber results in many solitons that could reach different wavelength shifts at the output, thus crowding the spectrum between 2.8  µm and the wavelength of the most shifted soliton, as seen in Fig. 7. Hence, one could think about using spectral filters to get rid of unwanted spectral contents. Ideally, to obtain a single soliton at the most shifted wavelength, we propose to insert two spectral filters in the system. The simulation results obtained with those two filters are shown in Fig. 13. The first filter is placed between the Er: $\hbox {ZrF}_4$ fiber and the $\hbox {InF}_3$ fiber. It is a high-pass wavelength filter ($\lambda > 3.05$  µm) that blocks all the amplifier generated solitons except the first one. The middle graph in the figure shows the output in the presence of this filter. The output is then composed of the results of the soliton fission of the first amplifier soliton. We thus have a soliton at 4.50  µm, followed by a soliton at 3.78  µm and a pulse remnant around 3.1  µm. All the spectral contents associated with the fission of the second amplifier soliton are now absent. In this manner, we make sure that no other soliton will reach the 4.5  µm region. Then, a second high-pass filter ($\lambda > 4.15$  µm) located at the output can single out the most shifted soliton generated by the fission of the first amplifier soliton, as shown in the bottom graph of the figure. In this case, we would thus have a clean pulse train of 4.5  µm hyperbolic secant pulses at 57.9 MHz.

Unfortunately, it was not possible to test this hypothesis in our system. The free-space filters that we could use introduced too much losses, significantly impairing the wavelength shift of the solitons. Moreover, no option for all-fiber spectral filters in the MIR seemed appropriate for this task at this moment. For instance, the use of chirped tilted fiber Bragg gratings^37^ which dump the rejected wavelengths toward the cladding could lead to an overheating of the polymer, damaging the fiber. The development of low-loss fiber-packaged spectral filters in fluoroindate fibers for the MIR could eventually enable this approach.

## Discussion

The proposed strategy to maximize spectral shifting relies on splicing a larger core fiber amplifier to a smaller core $\hbox {InF}_3$ fiber to create a high energy, higher-order soliton leading to soliton fission and enhanced wavelength shifting of the main soliton coming out from the fission process. This strategy can be seen as the fiberized equivalent of directly injecting a high-power seed pulse in the nonlinear fiber using free-space optics. However, an advantage of our technique compared to the direct free-space injection is that the amplifying fiber allows to pre-shift the main pulse while increasing its peak power, thus relaxing the requirements on the seed source. Hence, the same technique could be employed with lower seed pulse energy or longer pulse duration, given that the amplifier has enough pump power and/or fiber length to compensate. The same strategy can also be used at any wavelength where fiber amplifiers are available. This approach also prevents some of the difficulties associated with high-power free-space injection, such as thermal and mechanical stability issues. One drawback of this method is the generation of an overcrowded spectrum due to the presence of secondary solitons shifting to intermediate wavelengths. We have shown, however, that the use of cascaded spectral filters within the system could in principle lift this problem, although it still remains a challenging task from the technical viewpoint.

Although our system was designed to achieve the largest possible spectral shift, it could be modified to produce higher energy tunable pulses in a specific wavelength range depending on the targeted application. This could be achieved by adjusting the amplifier and passive fiber length or geometric parameters accordingly. For instance, an amplifier fiber with a larger core size could help to increase the soliton pulses energy, given that the reduced nonlinearity will allow the generated shifted solitons to remain in the gain band for an extended period. However, a larger passive fiber core would then also be required to avoid more splice losses and potential fiber damage at high powers, which will result in less spectral shift at the output.

It is also worth discussing the important spectral noise observed in the experimental spectra after the $\hbox {InF}_3$ fiber. One first note that although such noise was not really considered in most of the previously reported numerical simulations it was obviously plaguing other experimental results^28^. Now, some of these modulations can be attributed to the interference between closely spaced solitons, but the exact cause of this noise is still not perfectly understood. Notably, the noise could hardly be smoothed through averaging with the optical spectrum analyzer (OSA) and constantly varied in time, no matter the pump parameters or even the mode-locked seed source we used (several tests with alternative seed lasers were carried, yielding the same result). Since this noise arises mostly in the presence of the $\hbox {InF}_3$ fiber, we suspect it is related to the soliton fission dynamics. At the moment, it is hypothesized that slight fluctuations or accompanying remnants (e.g. Kelly sidebands) of the ring cavity seed output or, alternatively, Fresnel reflections from the fusion splice towards the gain media could induce chaotic-like shot-to-shot variations in the initial conditions of the soliton fission, but further efforts will be needed to confirm these hypotheses.

Finally, simulations enabled us to understand the main features of the signal dynamics in our system. In particular, they shed some light on the impact of soliton fission to enhance the wavelength shift. Nonetheless, our model could still be improved in numerous ways. First of all, a better knowledge of the fiber parameters would certainly help. For instance, the issue with the values of the transitions rates of the energy transfer upconversion and cross-relaxation processes in the Er:$\hbox {ZrF}_4$ fiber is not settled^38^. More data to determine the nonlinear parameters of the $\hbox {InF}_3$ fiber such as its nonlinear index $n_2$ and its fractional Raman contribution $f_R$ would be required. Also, in order to reproduce more precisely the experimental spectra and to address the problem of noise and stability of the spectrum, the model would have to be adapted to include a more realistic representation of the input signal (Kelly sidebands, fluctuations, etc.), a careful modelization of noise^39^ and maybe the inclusion of some feedback from different sections of the system.

## Conclusion and future work

We have shown that ultrashort pulses could be produced as far as 4.8  µm using SSFS and soliton fission inside an $\hbox {InF}_3$ fiber. We also delved in more details in the physical mechanisms at play allowing this large spectral shift, bringing light on the advantages of the method, but also its drawback regarding the spectral stability. Yet, several improvements can still be made to further simplify the experimental setup. Notably, using a fiberized pump combiner for the amplifier would reduce the number of free-space components. An all-fiber system can be imagined in the future given the realization of an all-fiber ultrafast laser in the mid-infrared. To avoid the spectral noise created by the soliton fission, an interesting alternative would be to use a conical shaped fiber to gradually increase the nonlinearity as the core size diminishes, therefore sustaining the SSFS over longer fiber distances. This would avoid the significant splice losses and most likely prevent soliton fission in the right conditions. In conclusion, we believe this technique could represent an interesting option to generate tunable ultrashort pulses in the MIR thanks to its relatively simple setup for many experiments and applications.

## Methods

### Fiber parameters

**Figure 14:**
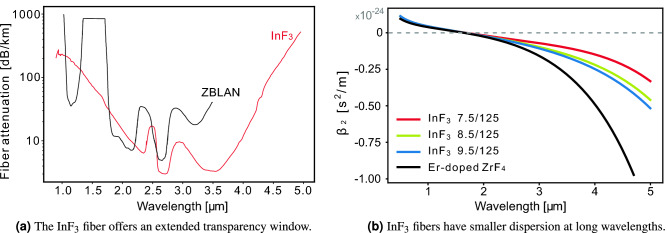
$\hbox {InF}_3$ fibers have better physical properties than ZBLAN for SSFS in the MIR.

The choice of an $\hbox {InF}_3$ fiber over a $\hbox {ZrF}_4$ fiber with the same dimensions is motivated by its extended transparency window, having losses of 1 dB/m around 5.4  µm versus 4.2  µm for the $\hbox {ZrF}_4$ fiber, and a lower anomalous dispersion at wavelengths higher than 3  µm which improves the SSFS process (see Fig. 14). Manufactured by *Le Verre Fluoré*, the 7.5/8.5/9.5  µm core fibers have a single-mode cutoff respectively at 2.95/3.3/3.7  µm. The Zero Dispersion Wavelength (ZDW) is around 1.65  µm and the numerical aperture (NA) is of 0.3. The $\hbox {ZrF}_4$-based double-clad doped fiber has a 15/240-260  µm core/cladding diameter with two parallel flats to improve the pump absorption. Its erbium concentration is of 7 mol%, a NA of 0.12 and has its ZDW around 1.65  µm and single-mode cutoff at 2.5  µm. Splice losses were of $\approx$ 40% for the three $\hbox {InF}_3$ fibers. Even though the mode mismatch was more important in the case of the 7.5  µm fiber, the experimental variation in splice quality accidentally compensated for this effect, thus resulting in similar losses for all three splices.

### Instruments and methodology

The spectra were obtained with a Yokogawa AQ6376 OSA operating between 1.9 and 5.5  µm. We used a resolution of 2 nm and the OSA was purged with $\hbox {N}_2$ to remove the $\hbox {CO}_2$ absorption lines. The spectra were measured directly at the end of the $\hbox {InF}_3$ fiber. A Gentec EO thermopile powermeter (XLP12-3S-H2) was used to measure the output power. The splice between the doped fiber and the $\hbox {InF}_3$ fiber was made using a Vytran GPX-3400 filament fusion splicer. The splice point was re-coated with low-index polymer while the end of the amplifier was re-coated with high-index polymer to evacuate residual pump (cladding mode stripper).

### Simulation model

The simulation model considers the single-pass propagation of the signal within the Er:$\hbox {ZrF}_4$ fiber followed by the $\hbox {InF}_3$ fiber. The fibers are treated as single-mode fibers even though the $\hbox {InF}_3$ single-mode cutoff is at 2.95  µm, slightly above some spectral contents of the input signal. The splice loss at the junction between the dissimilar fibers is calculated using modal overlapping^40^, considering the spectral dependence of the mode field diameters. The output spectrum is convoluted to represent the 2 nm resolution of the OSA in order to facilitate the comparison of the simulated and experimental spectra.

The signal considered at the input of the system is representative of the experimental data after injection losses are included, i.e. a pulse train of pure hyperbolic secant pulses at 57.9 MHz with a peak power of 1830 W, a full-width at half maximum pulsewidth of 440 fs and centered on a wavelength of 2.8  µm. For the results to be more representative, white noise with a bandwidth of 0.4  µm centered on 2.8  µm and having an average power of 30 mW over the full cavity roundtrip time is added to the input pulses. This white noise could represent different contributions, as discussed in the section “Discussion”.

The numerical propagation of the signal is based on the code presented in chapter 3 of the book *Supercontinuum Generation in Optical Fibers*^41^. This code takes into account the spectral dependence of the dispersive and nonlinear effects more accurately than the standard approach based on solving the nonlinear Schrodinger equation using the split-step Fourier method. This aspect is essential to reproduce the experimental results since during the soliton fission occurring in the indium fluoride fiber each soliton acquires a large bandwidth while remaining strongly localized. In the erbium-doped fiber, the model was adapted to include the gain by solving the steady-state populations of the erbium ions energy levels at the repetition rate of the laser source^34^.

**Figure 15:**
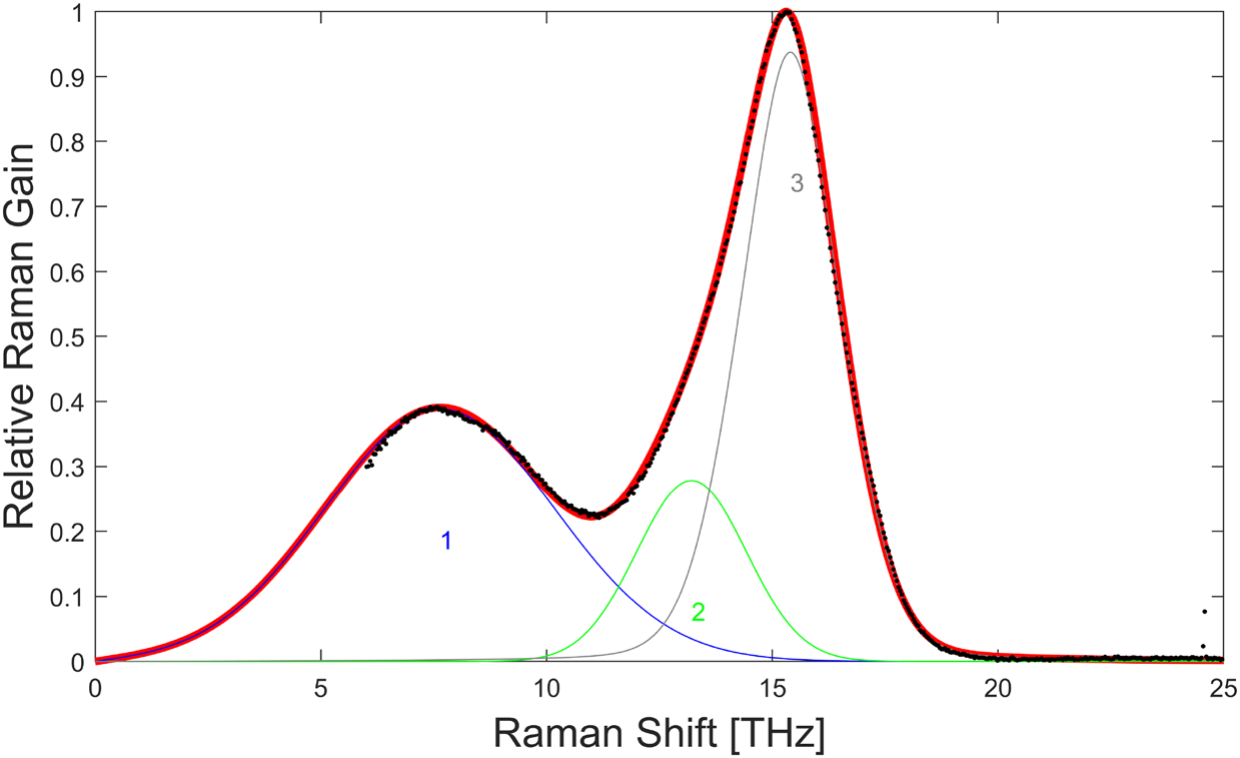
Relative Raman gain in $\hbox {InF}_3$ fiber: measurements (black dots) and curve fit (red) corresponding to the sum of 3 pseudo-Voigt profiles (blue, green, gray). An extrapolation toward small frequencies was required in the model.

The parameters of the Er:$\hbox {ZrF}_4$ fiber are given in our previous work^34^. However, instead of using the weakly interacting values for the transition rates of the energy transfer upconversion and cross-relaxation processes, we used the strongly interacting ones^38^, allowing a better fit of the experimental spectrum within the gain bandwidth. We also had to modify the power filling factor of the pump to $\Gamma _{p} = 0.05$%. For the $\hbox {InF}_3$ fiber, the losses as a function of wavelength were provided by *Le Verre Fluoré* and are shown in Fig. 14a. The dispersion parameters were obtained from a recent experimental characterization of the 7.5 µm indium fluoride fiber based on the use of an array of fiber Bragg gratings^42^ and shown in Fig. 14b. The nonlinear index is assumed to be $n_2 = 4.0\times 10^{-16}$ $\hbox {cm}^2$/W, lower than the bulk value of $4.3\times 10^{-16}$ $\hbox {cm}^2$/W given by Basaldua et al.^43^ but larger than their value of $3.2\times 10^{-16}$ $\hbox {cm}^2$/W measured in a fiber. The relative Raman gain spectrum of the fiber was measured with a MicroRaman spectroscopy system (Renishaw inVia) at 785 nm. The Raman gain was fitted with the sum of 3 pseudo-Voigt profiles with parameters $A_1 = 0.388$, $\eta _1 = 1$, $f_{01} = 7.63$ THz, $\Delta f_1 = 5.98$ THz, $A_2 = 0.278$, $\eta _2 = 1$, $f_{02} = 13.21$ THz, $\Delta f_2 = 2.86$ THz, and $A_3 = 0.938$, $\eta _3 = 0.8$, $f_{03} = 15.40$ THz, $\Delta f_3 = 2.72$ THz^34^, as shown in Fig. 15. The standard procedure was used to obtain the Raman transfer function. However, because the absolute value of the Raman gain is unknown, we assumed that the fractional Raman contribution was $f_R = 0.20$ to get a good fit with the experimental results. This value is comparable to the value of $f_R = 0.15$ in $\hbox {ZrF}_4$ fibers^34^. Finally, solving the characteristic equation for each fiber provided the effective index and the effective area of the mode and allowed the computation of the frequency dependent nonlinear coefficient^41^.

## Supplementary Information

Supplementary Information.

## Data Availability

The datasets used and/or analysed during the current study available from the corresponding author on reasonable request (Supplementary file).
