# Supplementary material for: Femtosecond tunable solitons up to 4.8 µm using soliton self-frequency shift in an InF3 fiber
Source: Sci Rep. 2022 Sep 23;12:15898. doi: 10.1038/s41598-022-19658-8 (PMC9508244; doi:10.1038/s41598-022-19658-8)

LE VERRE FLUORE
Rue Gabriel Voisin,Campus de Kerlann
35170 BRUZ - FRANCE
TEL : 02 99 05 31 30 FAX : 02 99 05 39 53

INSPECTION REPORT

200213/OF4408

**Fiber reference**	IFG SM [2.95] 7.5/125 - 10
**Part number**	200213/OF4408
**Core diameter**	7,5 µm
**Cladding diameter**	125 µm
**2nd Cladding diameter**	N/A
**Numerical aperture**	0.3
**Length**	10 m
**Doping ions**	N/A
**Concentration**	N/A
**Cut-Off wavelength**	2,95 µm
**Short term bend radius**	≥ 15 mm
**Long term bend radius**	≥ 45 mm

Attenuation curve

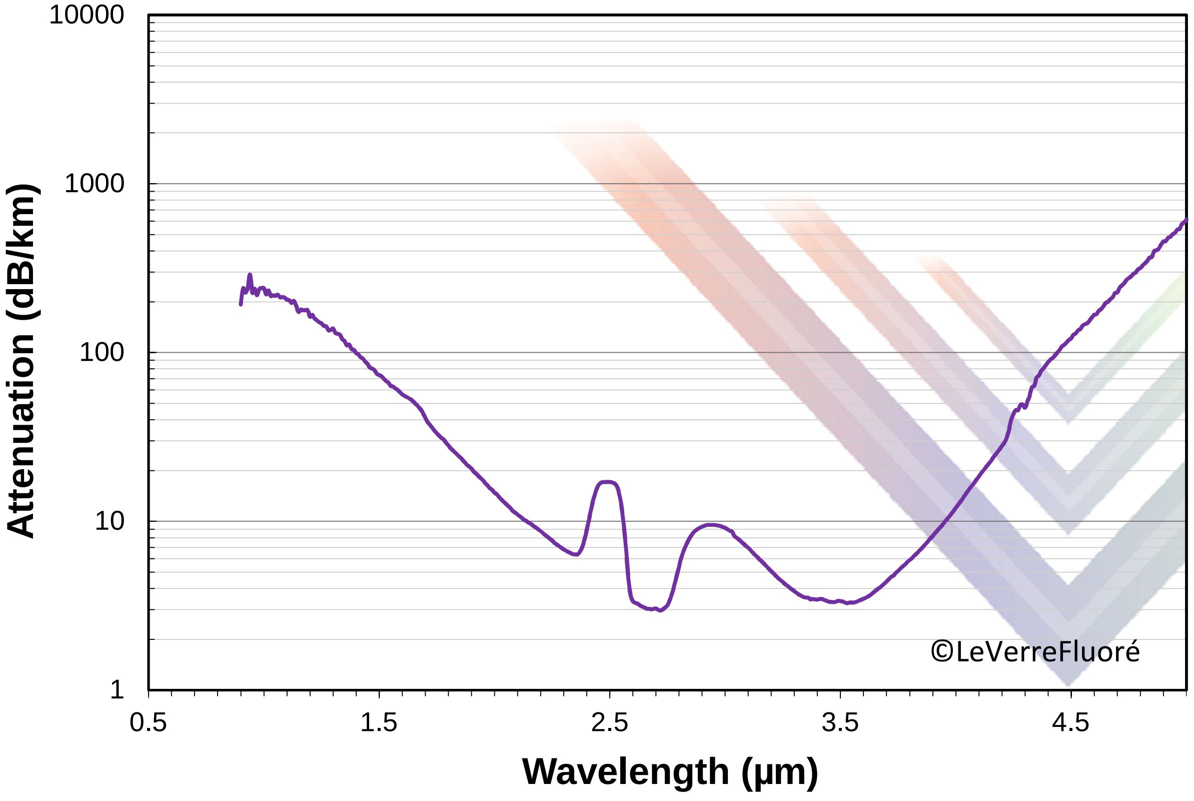

Supplement: Supplementary file 1 — Supplementary Information. [file 41598_2022_19658_MOESM1_ESM.zip › Raw data/Fig14a_FiberLosses/InF3 7.5um.pdf]
