# Supplementary figures and images for: Femtosecond tunable solitons up to 4.8 µm using soliton self-frequency shift in an InF3 fiber
Source: Sci Rep. 2022 Sep 23;12:15898. doi: 10.1038/s41598-022-19658-8 (PMC9508244; doi:10.1038/s41598-022-19658-8)

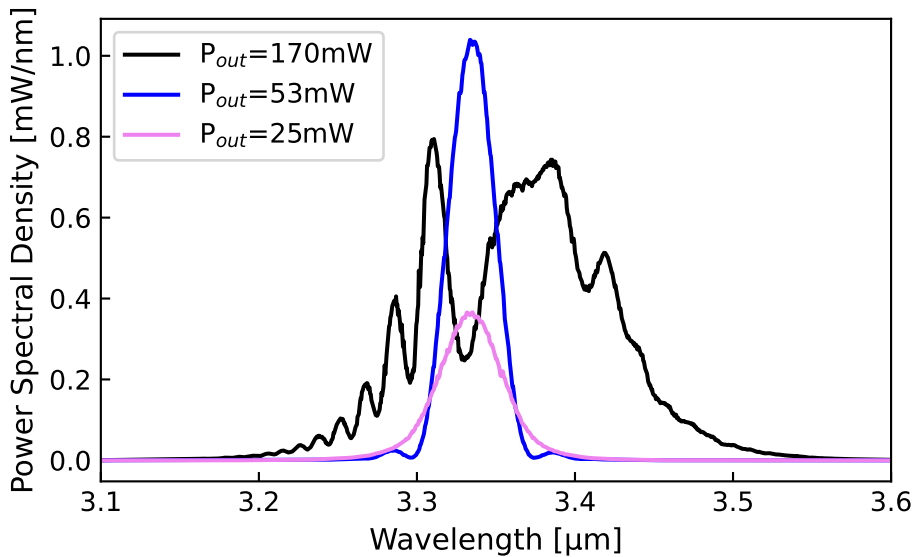

Supplement: Supplementary file 1 — Supplementary Information. [file 41598_2022_19658_MOESM1_ESM.zip › Raw data/Fig10_11/Fig10a.pdf]

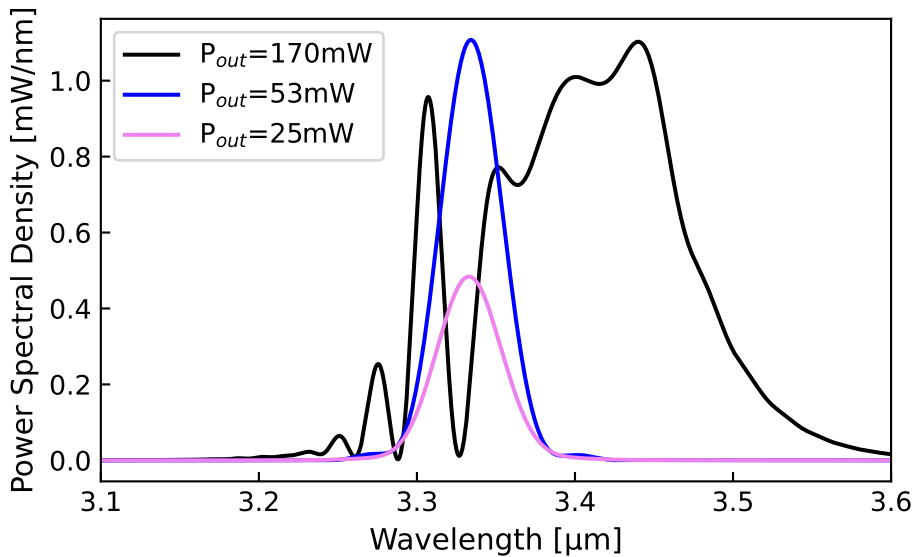

Supplement: Supplementary file 1 — Supplementary Information. [file 41598_2022_19658_MOESM1_ESM.zip › Raw data/Fig10_11/Fig10b.pdf]
